# Evolution of *VRN-1* homoeologous loci in allopolyploids of *Triticum* and their diploid precursors

**DOI:** 10.1186/s12870-017-1129-9

**Published:** 2017-11-14

**Authors:** Andrey B. Shcherban, Elena A. Salina

**Affiliations:** The Federal Research Center “Institute of Cytology and Genetics of Siberian Branch of the Russian Academy of Sciences”, Lavrentiev ave. 10, Novosibirsk, 630090 Russia

**Keywords:** Wheat, Vernalization, *VRN1* gene, Homoeoalleles, Allopolyploids, Promoter, First intron

## Abstract

**Background:**

The key gene in genetic system controlling the duration of the vegetative period in cereals is the *VRN1* gene, whose product under the influence of low temperature (vernalization) promotes the transition of the apical meristem cells into a competent state for the development of generative tissues of spike. As early genetic studies shown, the dominant alleles of this gene underlie the spring forms of plants that do not require vernalization for this transition. In wheat allopolyploids various combinations of alleles of the *VRN1* homoeologous loci (*VRN1* homoeoalleles) provide diversity in such important traits as the time to heading, height of plants and yield. Due to genetical mapping of *VRN1* loci it became possible to isolate the dominant *VRN1* alleles and to study their molecular structure compared with the recessive alleles defining the winter type of plants. Of special interest is the process of divergence of *VRN1* loci in the course of evolution from diploid ancestors to wheat allopolyploids of different levels of ploidy.

**Results:**

Molecular analysis of *VRN1* loci allowed to establish that various dominant alleles of these loci appeared as a result of mutations in two main regulatory regions: the promoter and the first intron. In the diploid ancestors of wheat, especially, in those of A- genome (*T. boeoticum*, *T. urartu*), the dominant *VRN1* alleles are rare in accordance with a limited distribution of spring forms in these species. In the first allotetraploid wheat species including *T. dicoccoides*, *T. araraticum* (*T. timopheevii*), the spring forms were associated with a new dominant alleles, mainly, within the *VRN-A1* locus. The process of accumulation of new dominant alleles at all *VRN1* loci was significantly accelerated in cultivated wheat species, especially in common, hexaploid wheat *T. aestivum*, as a result of artificial selection of spring forms adapted to different climatic conditions and containing various combinations of *VRN1* homoeoalleles.

**Conclusions:**

This mini-review summarizes data on the molecular structure and distribution of various *VRN1* homoeoalleles in wheat allopolyploids and their diploid predecessors.

## Background

Transition of plant from the vegetative stage to the generative one represents one of the most critical stages in the life cycle of a plant, since on its proper course depends: whether the plant gives the offspring. An active study of genetic mechanisms of transition to the stage of flowering on model plant species has made it possible to identify a complex network of genes that control this transition, depending on internal and external factors, such as temperature, length of daylight, hormone concentration, et cetera. The key genes of this system were identified and functionally characterized in the basic model object of plants, *Arabidopsis thaliana* (Reviewed in [1]). These genes can be divided into three types: 1) genes of regulatory cascades; 2) genes- integrators; 3) genes of the flowering meristems identity. Genes of the first type are conductors that transmit signals from separate external or internal factors to genes- integrators. The latter combine various regulatory cascades and, under a certain combination of factors, directly activate genes that induce the formation of flowering meristems.

In cereal plants, the duration of the vegetative period is determined by the interconnected systems of genes of vernalization response (*VRN1*, *VRN2*), response to photoperiod (*PHYC*, *PHYB*, *PPD1*, *CO1*, *CO2*), which trigger the genes of flowering meristems: *VRN1* and *LFY2* through the gene- integrator *FT1* (Reviewed in [2, 3]). *FT1* is a homolog of Arabidopsis *FT*, which encodes a mobile protein critical for the transmission of day-length signals from leaves to the shoot apical meristem [4]. A special role in this system is assigned to the *VRN1* gene, which combines the functions of a response to the vernalization signal and induction of the formation of flowering meristems from the apical cells of sprout. This gene encodes a MADS- domain containing transcription factor homologous to the product of the *AP1* gene of Arabidopsis [5]. Prolonged exposure to low temperatures (vernalization) induces the transcription of *VRN1* in both leaves and at the shoot apex [6, 7]. In the apex, *VRN1* initiates the transition of the shoot apical meristem to the reproductive stage whereas, in the leaves, it upregulates *FT1* directly [8], or through the *VRN2* gene [9]. Initially, deletion of the *VRN1* gene was thought to be the cause of irreversible loss of reproductive capacity in a radiation mutant in diploid wheat (*Triticum monococcum* L.), designated *maintained vegetative phase* (*mvp*) [6, 10]. However, a recent studies establish that, besides *VRN1*, some other genes, like *PHYC*, *VRN-Am1*, may be essential for wheat flowering raising the possibility that their deletion is responsible for the nonflowering phenotype of the *mvp* mutant [3, 11]. It should be noted that in allopolyploid cereals, including common hexaploid wheat *T. aestivum* L. (2n = 42; BBAADD), the genetic mode of the flowering regulation is greatly complicated due to the presence of homoeologous copies of each gene.

In common wheat the homoeologous loci *VRN-A1*, *VRN-B1* and *VRN-D1* were mapped on chromosomes of group 5 [12–14]. All three homoeologs are functional and their combination provides a predominant part of biodiversity of winter and spring forms of this species [15]. Winter wheat contains recessive alleles at each of these loci, while spring wheat, in at least one of them, has a dominant allele. The dominant allele *VRN-A1* causes complete insensitivity to vernalization and is epistatic with respect to the dominant *VRN-B1* and *VRN-D1* alleles, which cause little sensitivity to vernalization [16]. A wide range of the heading time in spring wheat is provided by various combinations of dominant alleles of the homoeologous *VRN1* loci that also influence the height of plants and yield [17–19]. The earliest in the time of heading are genotypes with three dominant alleles, however, they tend to be low-yielding. Genotypes with two dominant alleles (*VRN-A1*/*VRN-B1* or *VRN-D1*) ripen somewhat later and have higher yields. The presence of only one of the weak alleles *VRN-B1* or *VRN-D1* provides the latest ripening of spring varieties [16, 20]. The purpose of this mini-review is to analyze the available molecular genetical data on the formation of the *VRN1* allelic system at the following stages of wheat evolution: 1) in diploid donor species of A, B, G, and D genomes; 2) in tetraploid species (BBAA and GGAA genomes); 3) in hexaploid wheat (BBAADD).

### The main regulatory regions of *VRN1* loci affecting their expression

The molecular analysis of various *VRN1* homoeoalleles was first carried out, mainly, on polyploid wheat species as an object. It was shown that the dominant alleles of these loci are, mainly, associated with mutations in two basic regulatory regions: the promoter and the first (1st) intron [21, 22]. It is assumed that mutations in these regions disrupt interaction with regulatory factors leading to independent of ambient temperature, constitutive expression of *VRN1* in spring forms [2]. The dominant *VRN-A1* alleles associated with insertions (*VRN-A1a*) or deletions (*VRN-A1b*, *VRN-A1d*, *VRN-A1e*) in the promoter have been identified (Table 1). Initially, mutations in the CArG-like motif located 170 bp upstream from the starting ATG- codon were considered as the main determinants of the spring type [5]. However, later this motif was found to be invariant in a number of spring genotypes [23, 24]. Moreover, the complete deletion of the CArG-box retained sensitivity to vernalization. It was assumed that the CArG box can participate in response to photoperiod by binding a repressor protein expressed on a short day [25].

**Table 1:** Allelic variability in the promoter region and 1st intron of *VRN1* genes of wheat

Loci	Alleles	Status of regulatory regions/changes		Level of ploidy, genomes, species				References
		Promoter	1st intron	2n	4n		6n	
					BBAA	GGAA	BBAADD	
*VRN-A1*	*vrn-A1*	*vrn-A1*/ 8 bp insertion vs. *vrn-A1* or *VRN-A1* of all diploids	*vrn-A1*/no changes		*T. dicoccoides* *T. dicoccum* *T. durum*		*T. aestivum*	[21, 22, 26, 27, 29, 56]
	*vrn-A1u*	*vrn-A1u*/ SNPs vs. *vrn-A1* or *VRN-A1* of other diploids	*vrn-A1u*/ 1.4 kb deletion vs. *vrn-A1* or *VRN-A1* of other diploids	*T. urartu* (AA)				[27]
	*vrn-A1f*	*vrn-A1f*/ 50 bp deletion vs. *vrn-A1*	*vrn-A1*/ in some cases large indels were found (see below)			*T. araraticum, T. timopheevii*		[26.49]
	*vrn-A1f-del*	*vrn-A1f*/ 50 bp deletion	*vrn-A1f-del/* 2.7 kb deletion			*T. araraticum*		[49]
	*Vrn-A1f-ins*	*vrn-A1f*/ 50 bp deletion	*Vrn-A1f-ins/* 0.4 kb insertion			*T. timopheevii*		[49]
	*Vrn-A1f-del/ins*	*vrn-A1f*/ 50 bp deletion	*Vrn-A1f-del/ins /* 0.4 kb insertion, 2.7 kb deletion			*T. timopheevii*		[49]
	*Vrn-A1a*	*Vrn-A1a.1-a.3*/231 or 211 or 52 bp insertions	*vrn-A1*/no changes		*T. dicoccum* *T. durum*		*T. aestivum*	[21, 26, 29, 56]
	*VRN-A1b*	*VRN-A1b*/19 bp deletion	*vrn-A1*/no changes		*T. dicoccoides* *T. dicoccum* *T. durum*		*T. aestivum*	[21, 26, 27, 29, 56]
	*Vrn-A1c*	*vrn-A1*/no changes	*Vrn-A1c* / 5.5 kb deletion		*T. turgidum*		*T. aestivum*	[3, 21]
	*Vrn-A1d*	*Vrn-A1d*/ 19 and 32 bp deletions	*vrn-A1*/no changes		*T. dicoccoides*			[21, 26, 27]
	*Vrn-A1e*	*Vrn-A1e*/ 54 bp deletion	*vrn-A1*/no changes		*T. dicoccum* *T. durum*			[21, 26, 29]
	*VRN-A1f1*	*VRN-A1f1*/ 1 bp deletion	*vrn-A1*/no changes or *Vrn-A1ins*	*T. monococcum* (AA)				[25, 27]
	*Vrn-A1ins*	*vrn-A1*/no changes or *VRN-A1f1*	*Vrn-A1ins*/ 0.5 kb insertion	*T. monococcum* (AA)				[25, 27]
	*Vrn-A1g*	*Vrn-A1g*/ 34 bp deletion	*vrn-A1*/no changes	*T. monococcum*, *T.boeoticum* (AA)				[26]
	*VRN-A1h*	*VRN-A1h*/ 20 bp deletion	*vrn-A1*/no changes	*T.boeoticum* (AA)				[27]
	*Vrn-A1i*	*Vrn-A1i* / SNP	*vrn-A1*/no changes		*T. durum*			[29, 56]
	*Vrn-A1k*	*Vrn-A1k*/ 42 bp insertion	*vrn-A1*/no changes		*T. durum*			KX874608
	*Vrn-A1L*	*vrn-A1*/no changes	*Vrn-A1L* / 7.2 kb deletion		*T. dicoccoides* *T. carthlicum,* *T. polonicum* *T. durum*			[22, 27]
*VRN-B1/G1*	*vrn-B1*	*vrn-B1*/no changes	*vrn-B1*/no changes		*T. dicoccoides* *T. dicoccum* *T. durum*		*T. aestivum*	[21, 22, 26, 27, 29, 56]
	*vrn-B1sp*	*vrn-B1sp*/ minor changes vs. *vrn-B1*	*vrn-B1sp*/no large changes	*Ae. speltoides* (BB/GG)				[27]
	*VRN-B1dic*	*VRN-B1dic*/ 11% dissimilarity vs. *vrn-B1*	*vrn-B1*/no changes		*T. dicoccoides*			[28]
	*Vrn-B1a*	*vrn-B1*/no changes	*Vrn-B1a*/ 6.8 kb deletion		*T. durum*		*T. aestivum*	[22, 29, 56]
	*Vrn-B1b*	*vrn-B1*/no changes	*Vrn-B1b*/ *Vrn-B1a* + additional 36 bp deletion and SNP				*T. aestivum*	[57, 58]
	*Vrn-B1c*	*vrn-B1*/no changes	*Vrn-B1c* / 7.6 kb deletion, 0.4 kb duplication		*T. durum*		*T. spelta* *T. aestivum*	[34, 58, 56]
	*Vrn-B1ins*	*Vrn-B1ins*/ 5.4 kb insertion	*vrn-B1*/no changes		*T. carthlicum*			[53]
	*VRN-B1a*	*VRN-B1a*/ 127 bp insertion	*vrn-B1*/no changes		*T. turanicum*			[26]
	*vrn-G1a*	*vrn-G1a*/ 220 bp insertion	*vrn-B1*/no changes			*T. araraticum,* *T. timopheevii*		[26, 49]
*VRN-D1*	*vrn-D1*	*vrn-D1*/no changes	*vrn-D1*/no changes	*Ae. tauschii* (DD)			*T. aestivum*	[21, 22, 29]
	*Vrn-D1a*	*vrn-D1*/no changes	*Vrn-D1a* / 4.2 kb deletion				*T. aestivum*	[22]
	*Vrn-D1b*	*Vrn-D1b*/ SNP	*Vrn-D1a* / 4.2 kb deletion				*T. aestivum*	[63]
	*Vrn-D1с*	*Vrn-D1с*/ 174 bp insertion	*vrn-D1*/no changes				*T. aestivum*	[64]
	*Vrn-D1s*	*vrn-D1*/no changes	*Vrn-D1s* / 0.8 kb insertion				*T. aestivum*	[65]
	*Vrn-D(t)1*	*vrn-D1*/no changes	*Vrn-D(t)1*/ 5.4 kb deletion	*Ae. tauschii* (DD)				[50]

The so-called VRN box (“TTAAAAACCCCTCCCC”), directly 5′-adjacent to the CArG box [24], seems to have the greatest impact on the sensitivity to vernalization. Namely this motif is affected by insertions and deletions in the dominant alleles *VRN-A1a*, *VRN-A1d*, *VRN-A1e*, strictly associated with the spring type of growth [21, 26–28]. Recently, it has been established that even point mutations in the VRN box (the alleles *Vrn-A1b.3*, *Vrn-A1b.4* and *Vrn-A1i*) are sufficient for transformation into the spring type and the combination of these mutations can modulate the response to vernalization [29]. Upstream of the VRN box are binding sites for bZIP transcription factors (ACGT sites) [30, 31]. These sites can bind the TaFDL2 protein, which forms a complex with the product of the *VRN3* gene-integrator. It is this interaction that is supposed to provide the inducing effect of the *VRN3* gene on the transcription of *VRN1* [32].

Besides the *VRN1* promoter region, the region of the 1st intron also began to be considered as an important determinant of the spring habit after the discovery in wheat and barley of a number of dominant alleles containing only deletions in the 1st intron (the critical region is within 2.8 kb from the beginning of this intron) [22, 33, 34]. For example, the dominant alleles *VRN-A1c*, *VRN-B1a* and *VRN-D1a*, widely distributed in common wheat, contain large deletions in intron 1, whereas their promoter regions do not differ from those in the corresponding recessive alleles of these loci (Table 1). It was found that the repressed state of the *VRN1* gene observed in winter forms prior to vernalization is associated with a high level of methylation of histone H3 in the flanking region of the 1st intron and the degree of methylation is significantly reduced by deletions overlapping this critical region [35, 36]. An epigenetic model of the *VRN1* gene regulation was proposed, in which the 5′-flanking region of the 1st intron is attributed to the binding of chromatin modification factors affecting the transcription level of *VRN1* [37, 38]. According to the post-transcriptional model of regulation, this region contains a binding site for the Glycine-rich RNA-binding protein 2 (TaGRP2), which inhibits *VRN1* expression by binding with the *VRN1* pre-mRNA [39, 40].

An assessment of the relative level of transcription of various homoeoalleles *VRN1* was carried out using three near-isogenic lines Triple Dirk of *T. aestivum* bearing the dominant alleles *VRN-A1*, *VRN-B1* and *VRN-D1* [41]. An established higher level of transcription of *VRN-A1*, compared to two other homoeoalleles, leads to an earlier heading of the corresponding line. This may be due to the existence of several paralogs of this gene [42, 43] which may provide its overexpression, explaining the epistatic effect of *VRN-A1* in the digenic dominant *VRN1* genotypes (see above). It should be noted that differences in the level of transcription and phenotypic effect between dominant alleles of one homoeologous locus can be comparable with those between alleles of different loci. This was shown by Shcherban et al. [38] using near-isogenic lines containing *VRN-B1a* and *VRN-B1c* alleles. The revealed level of transcription of *VRN-B1c* was 10 times higher, probably, due to large structural changes (deletion and duplication) in the 1st intron compared with the first allele.

### The *VRN1* gene- associated prerequisites of spring growth habit in diploid precursors of wheat

Most of the wild Triticeae species both diploid and polyploid have a winter growth habit, suggesting that the recessive *VRN1* allele is the ancestral, original form [44]. Spring growth habit determined by dominant *VRN1* alleles could result from selection of mutations appearing during the adaptation of plants to environments that differ from the original. Some authors suggest that the spring type might have evolved from a previous winter plant prototype as an adaptation to warmer conditions, the feature that might be characteristic of both diploid and polyploid species [45, 46]. In support of this idea, Kato et al. [47] found that the distribution of spring forms of wild species *T. dicoccoides* Thell (2n = 28; BBAA) is sporadic and restricted to areas where vernalization is not required. Differentiation into spring and winter types was revealed in all *Triticum* and *Aegilops* diploid (2n = 14) species with the frequency of spring forms varying from 2% (*T. urartu*) up to 100% (*T. sinskajae*) [44].

In diploid species related to the A-genome (*T. boeoticum* Boiss., *T. monococcum* L., *T. urartu* Tum. ex Gandil.), the dominant *VRN1* alleles were most common among accessions of einkorn wheat *T. monococcum*, one of the earliest cultivated forms of wheat [48]. Shcherban et al. [27] revealed *Vrn-A1ins* allele (Table 1) in 35% of the studied accessions of *T. monococcum* and a total frequency of all dominant alleles was close to the percentage of spring forms (47%), established earlier for this species [44]. In the wild progenitor of *T. monococcum* - *T. boeoticum*, a few spring forms were associated with the *VRN-A1h* allele, although it was also found in some winter accessions (an optional type) [27]. The dominant *VRN-A1g* allele was quite rare in both *T. monococcum* and *T. boeoticum* [26]. The structure of regulatory regions of *VRN1* was highly conserved in the predominantly winter species, *T. urartu*, a donor of A-genome to polyploids. Interestingly, the latter species, in comparison with the other two diploids, has different mutations in both the promoter (nucleotide substitutions) and 1st intron (deletion of 1.4 kb) of *VRN1* providing specific identification of *T. urartu* and the corresponding A-genome in polyploids. However, all these mutations displayed no influence on vernalization requirements in all studied accessions, therefore the common *VRN1* allele of *T. urartu* is recessive (*vrn-A1u*; Table 1).

In *Ae. speltoides* Tausch, the presumable donor of B and G-genomes to polyploids, the structure of the promoter of *VRN-1* was more polymorphic compared to the *Triticum* A-genome species, however, most of this variation was upstream from the conserved region encompassing about 0.3 kb from the start codon and containing the putative regulatory sites [49]. No large indels were revealed in the *VRN-1* 1st intron of *Ae. speltoides.* All the studied accessions of this species were of winter type (allele *vrn-B1sp*; Table 1). Untill now, the dominant *VRN1* alleles associated with spring growth have not been found in *Ae. speltoides*.

The only dominant allele *VRN-D(t)1* was found in 9 out of 211 accessions of *Ae. tauschii* Coss., the proposed donor of D-genome to common wheat [50]. This allele contains deletion of a 5.4-kb sequence in the critical region of the *VRN1* 1st intron (see above) and was strongly associated with a lack of vernalization requirement (Table 1).

Thus, the dominant *VRN1* alleles within diploid species, presumable donors of A, B(G) and D-genomes, have a rare occurrence as compared with polyploid species (see below) that is consistent with the predominant winter type of these species. The only exception is *T. monococcum*, in which the spread of the dominant *VRN1* alleles and corresponding spring forms could occur due to the selection process carried out by human. This process resulted to a wide distribution of einkorn wheat from the Fertile Crescent to the more northern regions including the Caucasus, the Balkans, and central Europe [51].

### Polymorphism of *VRN1* loci in tetraploid wheat species of different origin

An important issue is the origin of the *VRN1* alleles associated with spring growth habit in the first tetraploid wheat species of two independent evolutionary lines: the Emmer line (BBAA) and the Timopheevii line (GGAA). The first line begins with the wild tetraploid *T. dicoccoides*, from which all the main cultural allopolyploids, including hard *T. durum* Desf. (BBAA) and common hexaploid wheat originated. The second line includes the wild species *T. araraticum* Jakubz. and derived from it, domesticated spring form- *T. timopheevii* (Zhuk) Zhuk*.*

Comparison of the *VRN1* alleles set of in the first tetraploid wheat species and those in the diploids shows that in the first tetraploids, the emergence of spring forms was associated with the appearance of new dominant *VRN1* alleles that are not related in origin to the dominant alleles of diploid predecessors (Table 1). The set of alleles of *T. dicoccoides* differs from that of Timopheevii tetraploids indicating an independent origin of the spring type of growth within these two branches of allopolyploids. In spring wheat, *T. timopheevii*, this trait is apparently associated with the insertion of a miniature transposon (MITE) in the 1st intron of the *VRN-A1* locus (alleles *Vrn-A1f-del/ins* and *Vrn-A1f-ins*; Table 1) [49, 52]. In *T. dicoccoides*, spring forms were associated with changes in the *VRN-A1* promoter: deletions of variable size (*Vrn-A1b*, *Vrn-A1d* and *Vrn-A1e* alleles), nucleotide substitution (*Vrn-A1i*) and a large deletion in the 1st intron (*Vrn-A1L*) [26–29]. Besides, after the divergence of both lines of tetraploids some changes in the *VRN-A1* promoter appeared to be not associated with spring growth habit including a 8 bp insertion in all Emmer wheats and a 50 bp deletion in Timopheevii wheats [49].

In contrast to *VRN-A1*, the *VRN-B1* locus in 83 accessions of *T. dicoccoides* displayed no significant indels, affecting the main regulatory sites and associated with the spring type [27]. The *VRN-B1dic* allele with a number of minor mutations within the promoter (SNPs, deletions up to 7 bp) was detected in one spring accession of *T. dicoccoides*, however, its influence on vernalization response has not been established definitely (Table 1).

In one accession of the Turanian wheat *T. turanicum* Jakubz. (BBAA) allele *VRN-B1a* (does not correspond to the dominant *Vrn-B1a*; Table 1) with an insertion in the *VRN-B1* promoter was found [26]. Interestingly, this insertion is homologous to the insertion in the *VRN-A1a* allele, however, it has other location- at position −100 from the start codon. The insertion of a retrotransposon of 5.4 kb in the *VRN-B1* promoter was detected in tetraploid Persian wheat *T. carthlicum* Nevski, and, unlike the previous case, its association with the spring forms was confirmed [53].

Besides *T. dicoccoides*, the *Vrn-A1L* allele containing large deletion in the 1st intron was identified in other Emmer tetraploid species of a later origin: *T. carthlicum*, *T. polonicum* L., *T. durum* [29, 54]. In *T. durum* the dominant *VRN-B1a* and *VRN-B1с* alleles appeared, having deletions of 6.8 and 7.6 kb, respectively, in the 1st intron (Table 1). The latter allele seems to have originated from *VRN-B1a* due to an additional deletion of 0.8 kb and a duplication of 0.4 kb [38, 55]. The both *VRN-B1* alleles are higly distributed among spring varieties of common wheat (see below).

The only identified mutation affecting *VRN-G1* locus in Timopheevii wheats is an insertion of foldback element in the promoter region at position −99 from the ATG-codon (allele *VRN-G1a*) [26]. However, in fact this allele is recessive since it was later found in winter accessions of *T. araraticum* [49].

Thus, in the first allotetraploid wheat species of both Emmer and Timopheevii lines the most variability affected the locus *VRN-A1*, while the other homoeolog- *VRN-B1* (*G1*) remained unchanged in spring forms (*T. dicoccoides*), or mutation in it did not result to the spring phenotype (Timopheevii, allele *VRN*-*G1a*). On a later stages of evolution of Emmer allotetraploids, especially, in the cultivated *T. durum*, different *VRN-B1* alleles appeared containing mutations in the promoter or 1st intron regions.

### Variability of *VRN1* loci in hexaploid wheat

Many known *VRN1* alleles, common in hexaploid wheat, originated at the tetraploid stage. This refers to such alleles as *VRN-A1a*, *VRN-A1b*, *VRN-A1c*, *VRN-B1a*, *VRN-B1c* [21, 22, 26, 29, 55–58]. The most ubiquitous among them are the *VRN-A1a* allele with a foldback insertion in the VRN-box and the *VRN-B1a* allele. Simultaneously, a number of a new alleles including *VRN-B1* (*VRN-B1b*) and, especially, *VRN-D1* (*VRN-D1a*, *VRN-D1b*, *VRN-D1c*, *VRN-D1s*) loci appeared in hexaploid wheat (Table 1).

The *VRN-B1b* allele appears to have originated from the *VRN-B1a* allele, since along with large deletion in the 1st intron, characteristic of the latter, it has an additional SNP and a 36 bp deletion [57]. This allele has been detected in common wheat originating from North America and was associated with the spring growth habit [58].

The dominant *VRN-D1a* allele was first isolated from spring near-isogenic line TDE (Table 1). As shown, *VRN-D1a* is the predominant allele in spring wheat genotypes adapted to tropical and subtropical regions [59–62]. The *VRN-D1b* allele has been originated from the previous allele due to SNP in the CArG-box at the promoter region [63]. Since the F2 population plants with *VRN-D1b* headed 32 days later than those with *VRN-D1a*, the authors suggest that a single nucleotide mutation at promoter region may modify the basal activity level of an allele of *VRN1* that is already active (due to the loss of segment in intron 1). The *VRN-D1c* allele with 174-bp insertion in the promoter region was discovered in three out of 205 Chinese wheat cultivars [64]. As suggested, the insertion in *Vrn-D1c* may contribute to the increase in gene expression and cause early heading and flowering due to transcriptional cis-elements. In the same year Muterko et al. [65] found the *VRN-D1s* allele resulted from DNA transposon insertion of 844 bp in the 1st intron and associated with spring form (Table 1).

It can be assumed that the wide distribution of spring forms and associated with them combinations of *VRN1* homoeoalleles in common wheat is largely due to artificial selection directed to adaptation of wheat to different climatic conditions. As one of the illustrations, the analysis of distribution of different *VRN1* haplotypes among varieties of spring common wheat from different eco-geographical regions of Europe and Russia can be presented [34, 59]. For the group of cultivars of the northern and central Europe and the most of territory of Russia, the digenic dominant haplotypes at the *VRN-A1* and *VRN-B1* loci are characteristic, while for the southern European group of cultivars, the monogenic dominant haplotypes containing either the dominant *VRN-B1* or *VRN-D1* are mostly widespread. The latter group of cultivars had a later date of heading compared to the cultivars of the first group. Therefore, the monogenic *VRN-B1*/*VRN-D1* haplotypes could have a breeding advantage in the subtropical southern regions, providing a longer vegetative period that is most effective for this zone in terms of yield. The early ripening, digenic dominant haplotypes, in turn, have an advantage in regions with a temperate climate, where there is a risk of early fall frosts [59].

## Conclusions

In the diploid predecessors of polyploid wheats, the dominant *VRN1* alleles associated with spring forms have a limited distribution, mainly, in a specific climatic regions where such forms may receive a natural selective advantage over winter forms. The origin of spring forms in polyploids, starting from the first wild-growing tetraploid species (BBAA/GGAA) is due to emergence and spread of a new dominant *VRN-1* alleles, not related by origin to alleles of the diploid species, presumed donors of A-, B (G) and D- subgenomes. In cultivated wheat polyploids, especially in common wheat, the process of artificial selection significantly enhance the spread of different *VRN1* alleles at each of the homoeologous loci and their combinations that determine the optimal heading time for a district geographical regions.
